# Prosthetic Valve Endocarditis due to *Kytococcus schroeteri*

**DOI:** 10.3201/eid0911.020683

**Published:** 2003-11

**Authors:** Karsten Becker, Jörg Wüllenweber, Hans-Jakob Odenthal, Michael Moeller, Peter Schumann, Georg Peters, Christof von Eiff

**Affiliations:** * University Hospital of Münster, Münster, Germany; † University Hospital of the Saarland, Homburg/Saar, Germany; ‡ Mathias-Spital, Rheine, Germany; § German Collection of Microorganisms and Cell Cultures, Braunschweig, Germany

**To the Editor:** Bacteria belonging to the former genus *Micrococcus*, the so-called micrococci, are usually regarded as contaminants from skin and mucous membranes. Nevertheless, micrococci have been reported as emerging pathogens in immunocompromised patients and have been described in severe infections (*1*–*4*). We describe what is, to our knowledge, the first case of prosthetic valve endocarditis caused by the newly described micrococcal species, *Kytococcus schroeteri*. Accurate identification of this species is of particular importance as kytococci—in contrast to other micrococcal species—are frequently resistant to penicillin and oxacillin (*5*).

A 34-year-old woman was admitted to the hospital with acute, severe aortic regurgitation, attributable to a dissection of both the ascending and descending aorta, which extended into the supraaortic and iliac arteries. Immediate surgical intervention was performed by implantation of an aortic arch (St. Jude Medical Inc., St. Paul, MN) conduit and reimplantation of the supraaortic arteries. Ten weeks later, the patient was admitted to the hospital because of fever of 39°C. Laboratory studies showed a leukocyte count of 15.3 x 10^9^/L with 87% neutrophils and elevated C-reactive protein (180 mg/L). Transesophageal echocardiography and computed tomography suggested an abscess next to the prosthesis and showed vegetations on the prosthetic valve, which suggested endocarditis. Blood cultures yielded gram-positive cocci on four separate occasions during an 11-day period. Treatment, performed according to the antimicrobial susceptibilities of the isolates, consisted of vancomycin, gentamicin, and rifampin for 21 days. Within 1 week, the fever resolved and the leukocyte count returned to normal. Four days after antimicrobial therapy was initiated, right-sided hemiparesis and aphasia, thought to be due to an embolic cerebral stroke, developed. After those events, the aortic arch prosthesis was replaced without further complications.

Blood culture specimens were injected into BACTEC Plus culture vials for aerobic and anaerobic cultures and processed in BACTEC 9240 blood culture system (Becton Dickinson, Cockeysville, MD). Growth was detected in four different aerobic blood cultures after incubation of 3 to 5 days. Aerobic subcultures on Columbia agar supplemented with 5% sheep blood showed tiny, muddy-yellow colonies without hemolysis after 24 h of incubation. After 48 h, the size of colonies increased, a feature typical of *K. sedentariu*s, which is known to grow slightly more slowly than other members of the former *Micrococcus* genus. No or very weak reactions were found after 24 h incubation when the ID32 STAPH ATB gallery (bioMérieux Vitek, Hazelwood, MO) was used. After 48 h, the reactions with this gallery resembled those of *M. luteus* or *M. lylae.* The probability of identification was indicated as 99.0% (*M. luteus*, T index of 0.77) for the profile 000003000 and 51.8% (*M. lylae*, T index of 0.98) and 47.2% (*M. luteus*, T index of 0.93), respectively, for the profile 000001000. When the ID-GPC card (VITEK 2, bioMérieux Vitek) was used, a poor selectivity was observed (*M. luteus*, T index 0.95; *Kocuria rosea*, T index 0.84). All isolates were resistant to oxacillin, penicillin, fosfomycin, ampicillin, and erythromycin and susceptible to vancomycin, teicoplanin, gentamicin, netilmicin, chloramphenicol, imipenem, rifampin, tetracycline, amoxicillin/clavulanate, and ciprofloxacin, as determined by disk diffusion method performed on Mueller-Hinton agar.

When arbitrarily primed-polymerase chain reaction with prolonged ramp times (*6*) was used, isolates were shown to be clonal, representing one strain (DSM 13884^T^). Since colony formations, resistance pattern, and growth rate of this strain did not correspond with the species identification, as obtained by automated systems, further phenotypic and molecular studies were conducted, confirming the micrococcal nature of this unknown strain and justifying the classification as a distinct species, *Kytococcus schroeteri* sp. nov (*7*).

In addition to the *Micrococcus* genus, bacteria belonging to the former genus *Micrococcus* were recently divided into the genera *Kocuria*, *Nesterenkonia*, *Kytococcus*, and *Dermacoccus,* followed by rearrangement into two families (*Micrococcaceae,* *Dermatophilaceae*) of the suborder *Micrococcineae* (*5*).

The traditional identification of the micrococci is based on their susceptibility to lysozyme and bacitracin and their resistance to lysostaphin and nitrofurantoin, in contrast to staphylococci, which display the opposite pattern. In automated identification systems, micrococci are included only in a limited manner. A prospective study showed an overall accuracy of results of 61.0% concerning *Micrococcus* species when the STAPH-IDENT strip (bioMérieux) was compared with conventional identification methods (*8*).

Micrococcal species are ubiquitous inhabitants of the human skin and mucous membranes and are usually disregarded as contaminants in clinical specimens. Yet, various severe infections such as arthritis, central nervous system infection, pneumonia, peritonitis, hepatic abscess, endocarditis, and nosocomial blood stream infections have been documented (*1*,*3*,*4*,*9*). Since early reports of endocarditis caused by gram-positive cocci that appear in tetrads and packets often did not reliably differentiate between micrococci and phenotypically similar microorganisms, such as coagulase-negative staphylococci, the frequency of micrococcal endocarditis is difficult to ascertain and might be underestimated. However, several cases of endocarditis attributable to *M. lylae*, *M. luteus*, *K.* *sedentarius,* and unspecified micrococci have been reported (*1*).

Regarding micrococci, data on antimicrobial susceptibilities are rare, and often the species affiliation remains unclear. In contrast to most micrococcal isolates, *K. sedentarius* isolates, as well as those reported here, are resistant to penicillin G and oxacillin. In the patient we describe, therapy was performed with vancomycin, gentamicin, and rifampin, resulting in bacteriologic eradication and clinical cure. However, a generally accepted therapeutic regime for severe infections with kytococcal species has not yet been defined. Concerning micrococci other than kytococci, a combination of rifampin with ampicillin has been effective (*3*). Successful treatment has also been achieved with vancomycin, clindamycin, penicillin, gentamicin, or a combination of these agents. Overall, rifampin shows the highest activity against all micrococcal species (*10*).

This report is the first case of *K. schroeteri* causing endocarditis on an artificial heart valve. The repeated recovery of this species from blood cultures strongly suggests a pathogenic role. We conclude that isolation of micrococci from blood specimens cannot always be disregarded as etiologically irrelevant. Results performed by automated identification systems should be interpreted with caution if micrococci are involved.
